# An Act to Regulate the Licensing of Practitioners of Physic or Surgery

**Published:** 1883-04

**Authors:** 


### An Act to Regulate the Licensing of Practitioners of Physic or Surgery.

We are able to present our readers the act proposed by the Committee on Legislation of the State Medical Society, which it is intended to present to the legislature. In our last number we urged the importance of this matter with a view to the general interests of the profession and the public. The following act covers the ground very fully, and, with some slight modifications, will receive the endorsement of the profession. We invite our readers carefully to peruse it.

*The People of the State of New York represented in Senate and Assembly do enact as follows:*

Sec. I.—The Regents of the University of the State of New York shall appoint as many State boards of examiners in medicine as they shall deem necessary, the membership of one or more of which boards shall represent either one of the several systems of medical practice recognized by the incorporated State medical societies of this State, each board to consist of not less than nine members, who shall be legally authorized practitioners of physic or surgery in this State; and all persons desiring to practice physic or surgery after November first, eighteen hundred and eighty-three, must comply with the provisions hereinafter prescribed, and obtain the license hereinafter provided before entering upon the practice of physic or surgery in this State.

SEC. II.—Such examiners shall faithfully examine all candidates referred to them for that purpose by the Chancellor of said University and furnish him a detailed report in writing of all of the questions and answers of each examination, together with a separate written opinion of each examiner as to the qualifications and merits of the candidate in each case.

SEC. III.—Such examination, upon questions furnished by the Regents of the University of the State of New York, shall be in Anatomy, Physiology, Histology, Pathology, Theory and Practice of Medicine, Chemistry, Surgery, Obstetrics, Materia Medica and Therapeutics, and such other branches in the several departments of medical science as may be required from time to time by the said Regents.

SEC. IV.—The said reports of the examinations shall forever be a part of the public records of said University, and the orders of the Chancellor addressed to the examiners together with the action of the Regents in each case shall accompany the same.

SEC. V.—Any person on paying not less than twenty-five dollars into the treasury of the University, and on applying to the Chancellor for the aforesaid examination, shall receive an order addressed to one of the aforesaid boards of examiners, instructing it to examine the candidate at one of the regular semi-annual examinations, provided that proof satisfactory to the Chancellor is first given, that the candidate is over twenty-one years of age, of good moral character, and has received a diploma issued to him or her and conferring upon him or her the degree of Doctor of Medicine from some legally incorporated Medical College.

SEC. VI.—The Regents of the University, after finding that not less than three-fourths of the members of one of said boards have voted in favor of a candidate, and that said examination has been a satisfactory test of the qualifications of said candidate, shall issue to him or her a license to practice physic or surgery in the State of New York, for which license the candidate shall pay to the University the further sum of not less than fifteen dollars.

SEC. VII.—The moneys paid to the University under the provisions of this act shall be appropriated by said Regents for defraying the expenses incurred by reason of the provisions of this act.

SEC. VIII.—The Regents shall establish such rules and regulations as they may deem necessary to insure the faithful execution of this act.

SEC. IX.—Every person (excepting such as have heretofore lawfully registered pursuant to the laws of the State in force at the time) complying with Sections V and VI of this act, shall, before commencing to practice physic or surgery, register in the clerk's office in the county where he or she practices or intends to practice physic or surgery, in a book to be kept by the said clerk, his or her name, residence, and place and date of birth, together with the date of his or her diploma, and by what institution granted, together with his or her license to practice physic or surgery within this State; at the same time the person so registering shall exhibit both the license and the diploma herein required to the clerk, and subscribe and verify by oath or affirmation before a person duly qualified to administer oaths under the laws of this State, an affidavit containing a plain statement of all of the facts as aforesaid, including his or her age, and shall file said affidavit and copy of said diploma with said clerk; said county clerk to receive a fee of twenty-five cents for such registration, payable by the person so registering. Nothing in this section shall be so construed to prohibit medical consultations in the different counties of this State between legally qualified and registered physicians of this and neighboring States.

SEC. X.—A person who shall willfully swear falsely to any statement contained in the affidavit required by the ninth section of this act, shall be deemed guilty of and subject to conviction and punishment for perjury, and a person who violates any of the other provisions of this act, or who shall practice physic or surgery under cover of a diploma unlawfully issued or illegally obtained or without a license as provided for in this act, shall be deemed to be guilty of a misdemeanor, and on conviction thereof shall be punished by a fine of not less than two hundred and fifty dollars, nor more than five hundred dollars for the first offense, and for each subsequent offense by a fine as aforesaid and by imprisonment for not less than thirty days and not more than six months. The fine, when collected, shall be paid, one-half to the person or the corporation making the complaint, the other half into the county treasury.

SEC. XI.—For the purpose of this act the words practice physic or surgery, shall mean to annex the letters M. D. to one's name, or to suggest, recommend, or prescribe, direct or employ, as a matter of business or for a fee, for the use of any person any drug, medicine, appliance, apparatus, or other agency, whether material or not material, for the treatment, cure, relief or palliation of any real or supposed ailment or disease of the mind or body, or for the treatment, cure, relief, of any wound, fracture, or other bodily injury or infirmity or any deformity.

SEC. XII.—Nothing in this act shall apply to commissioned medical officers of the United States Army or Navy, or of the United States Marine Hospital service during the term of their service as such, nor to any of the members of the House Staff of any legally incorporated Hospital during the term of service in the said Hospital.

SEC. XIII.—So much of chapter seven hundred and forty-six, laws of eighteen hundred and seventy-two; chapter five hundred and thirteen, of laws of eighteen hundred and eighty; chapters eighty-six and six hundred and seventy-nine, laws of eighteen hundred and eighty-one, and all other acts and parts of acts inconsistent with this act are hereby repealed.

SEC. XIV.—This act shall take effect November first, eighteen hundred and eighty-three.

